# PICI: A web server with a multi-parametric algorithm for identifying interaction sites within protein complexes

**DOI:** 10.6026/97320630012078

**Published:** 2016-04-10

**Authors:** Arindam Atanu Das, Ramadas Krishna

**Affiliations:** 1 Centre for Bioinformatics, Pondicherry University, Puducherry 605014, India

## Abstract

Identifying interactions between proteins in a complex is essential to analyze their role in various cellular activities and structural stability. Therefore, effective algorithms and computer programs are required for the better understanding of various interactions exist at the protein-protein interface. The protein inter-chain interaction (PICI) server was developed to identify these interaction sites and various interactions that contribute to the specificity and strength of the protein complex by using Protein Interaction Identification Algorithm (PIIA). The multi-parametric approach of this algorithm involves the interface area between the subunits, the atomic coordinate distance, the linearity of bonds, the orientation of aromatic side-chains, and physicochemical properties of the residues. Particular advantages of PICI include its ability to identify various strong and weak interactions, including those which have not been considered before by any other server. Representation of interface data in text tables, 3D interaction visualizer, colored sequence viewer, and a dynamic colored graphical matrix display makes it distinct from similar web servers. Users can analyze interactions within multi-chain proteins by their PDBids or by uploading a structure atomic coordinate file and can download the result in plain text or XML format.

**Availability:**

PICI is freely accessible at http://pici.bicpu.edu.in/

## Background

Analyzing interactions within protein complexes are crucial to gain a better understanding of their role in signaling pathways, enzyme regulation, receptor binding, antigen recognition, and several other cellular processes, hence are prime targets for drug design. Earlier studies have described the principles governing the interactions and factors that influence the formation of protein complexes [1]. Many papers have discussed different kinds of protein interactions such as disulphide bonds [2], interaction between hydrophobic residues [3], hydrogen bonds [4], electrostatic interactions [5], aromatic-aromatic [6], cation-π [7], anion-π [8], and atoms having lone pair electrons (N, O, S) to π interactions [9-10]. Along with these interactions several other hydrogen bond weaker than classical hydrogen bonds are also present in proteins, such as C-H···N, C-H···O, C-H···S, C-H··· π, N-H··· π, O-H··· π, and S-H··· π bonds [4, 11,12,13]. PPI is largely governed by numerous such week interactions. Here we report a web server PICI (Protein Inter-Chain Interactions) version 3.2, which calculates and recognizes various kinds of interactions in structural coordinate files of proteins. PICI initially developed as an integrated tool in PepBind database server [14] to aid analysis of protein-peptide interaction interface. The improved web server includes several programs and scripts to execute its function more efficiently across large protein-protein interfaces.

## Methodology

### Architecture and Data Processing

**Figure 1:**
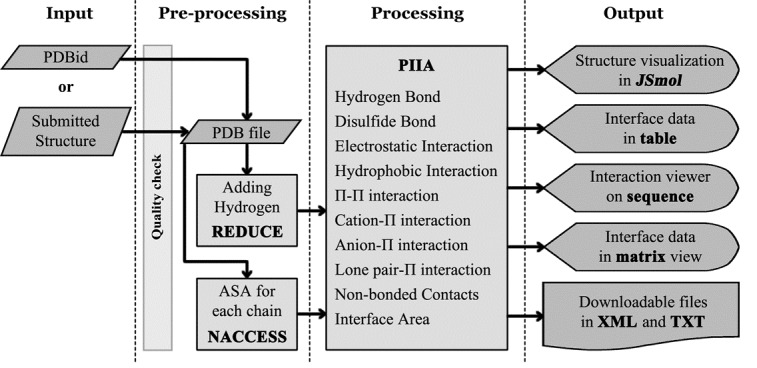
Dataflow diagram of PICI server.

PICI computes all strong and weak interactions using atomic coordinate data structure files (PDB format) with various distances and angle parameters. The data flow and processing of the web server pass through four stages: (i) Input, (ii) Preprocessing, (iii) Processing with PIIA, and (iv) Output (Figure 1). For calculation of interface area, PICI processes each chain within the multi-chain protein files separately using NACCESS [15] program with 1.4Å probe (radius of a water molecule). The REDUCE [16] algorithm is used to precise the position of hydrogen atoms in the structure file. The hydrogen added file is then parsed for the atomic coordinate positions and processed in PIIA program to identify the interactions.

### PIIA(Protein Interaction Identification Algorithm)

PIIA calculates nine different types of interactions between neighboring chains based on (i) the interface area, (ii) the atomic coordinate distance, (iii) the linearity of bonds, (iv) the orientation of aromatic side chains, and (v) the physicochemical properties of the residues. The integration of these methods enables PICI to offer efficient identification of location and types of interactions with greater reliability of results. Default cutoff distance and angle for calculations are shown in Table 1. Users can also define their preferred parameters for each type of interactions.

### PIIA considers the following criteria for calculation

(a) For NMR structures, only the first model is considered; (b) Cysteine gamma sulfur atoms which participate in disulfide bridges are not considered as proton donors while calculating hydrogen bonds; (c) Backbone nitrogen atoms (except terminal NH2) are excluded from proton acceptors due to delocalization of lone pair electrons with the neighboring carbonyl group; (d) Hydrophobicities of residues are based on solvent transfer free energies from octanol to water [3]. (e) Both 6 and 5 member rings of tryptophan side-chain are considered as separate π- rings for aromatic interactions; (f) Histidine is considered to be in neutral form and side chain imidazole as an aromatic motif; (g) The ammonium nitrogen (NZ) in Lysine and guanidinium carbon (CZ) in Arginine are considered as cationic charge centers for cation–π interaction [7].

### PICI Web Interface

**Figure 2:**
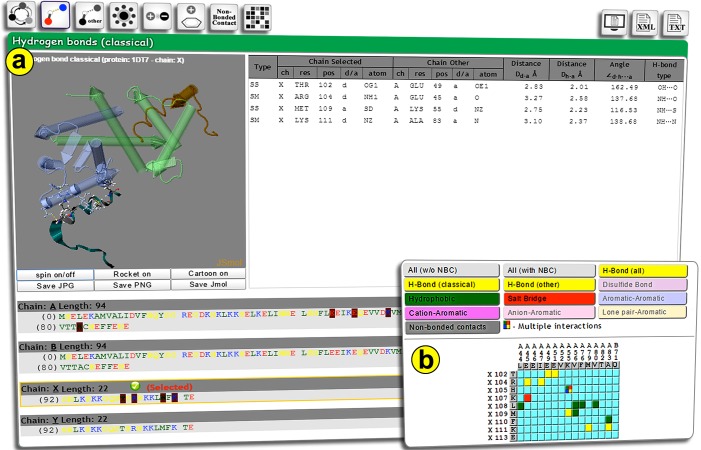
Snapshots of the PICI server result viewer page depicting the interface between the two subunits in a protein (PDBid: 1DT7). The top panel shows buttons for all interaction types along with download links for XML and TXT files: (a) Screenshot of interaction result page showing three sections; 3D interface visualization with JSmol, interaction information in a table, and highlighted interacting residues on sequence; (b) Screenshot of the matrix for all interactions, excluding non-bonded contacts with colored compartments for each type of interactions.

PICI web interface is developed with a set of PHP scripts and JavaScripts to display the generated data interactively by responsive HTML web pages with CSS, which work on all major web browsers. PICI takes a protein structure file in PDB format as input. After pre-processing and processing the input data, it displays the output in an interactive result webpage. On the result viewer page, each button on the top panel corresponds to specific interactions and shares a common web page composed of three panels (structure viewer, sequence viewer, and simple text tables) to facilitate the analysis of the information (Figure 2a). PICI also generates two output formats: TXT (plain text) and XML (Extensible Markup Language) files to be downloaded by users.

### 3D interaction viewer panel

The structure visualization panel dynamically displays interacting residues in the JSmol structure viewer which is compatible with all browser types. It allows users to visualize atomic details of all interacting residues at the interface.

### Sequence viewer panel

Amino acid sequences of the multi-chain protein are shown and residues are highlighted for each interaction. The single letter code residues are colored by their physicochemical properties such as green for hydrophobic, yellow for neutral, red for negatively charged, and blue for positively charged residues. The interacting residues in the sequence are dynamically highlighted for each type of interaction. BLAST links are generated for each sequence to perform sequence similarity search on PDB or UniProt database.

### Interaction matrix

PICI also generates a dynamic matrix of interacting residues in the selected chain to other chains with colored compartments (Figure 2b). Each color represents an interaction type, such as yellow for hydrogen bond, purple for disulfide bridge, red for the salt bridge, green for hydrophobic, blue for π-π, violet for cation-π, pink for anion-π, orange for lone pair-π interactions, and gray for non-bonded contacts. The compartments, which show multiple interactions, are represented by multiple colors. The matrix representation of interaction provides an overall view of the protein-protein interface graphically.

## Discussion

PICI web-server will serve as a useful resource for the structural bioinformatics and the proteomics community to perform analysis of protein structures based inter-chain interactions. Although there are some existing web servers doing similar jobs such as PIC [18], PDBsum (Protein-protein interface) [19] and PISA (Interface) [20] for protein interactions, they target some specific research area and have their unique data representations. PICI server is unique in calculating nonclassical hydrogen bonds, anion-π, and Lp-π interactions, which are being ignored by other existing protein interaction algorithms. PDBsum and PISA show only donor-acceptor distances for hydrogen bonds without showing their bond angles. Although PIC calculates the hydrogen bond angle, it does not consider bond linearity as a criterion for a hydrogen bond. Also, PIC server does not calculate the interface area and non-bonded contacts between residues in close proximity. The uniqueness of PDBsum’s protein-protein interface program lies in its schematic colored diagram of inter-chain interactions. PISA interface is unique in its ability of deep analyses of PPI interface and informative colored representation of the interface data table. A newly developed PIMA server [21] is unique in data presentation with a dot plot for interaction energies, but it is also limited to few interaction types. PICI’s PIIA program is unique in analyzing protein-protein interactions based on multiple structural and physicochemical parameters. Data representation in PICI is unique in presenting the interaction data in (i) highlighted interacting residues in a colored sequence viewer, (ii) color coded interaction matrices, (iii) Java independent structure viewer, and (iv) a textual data table. (A comparative analysis of different web servers along with example are given in supplementary documents).

## Figures and Tables

**Table 1:** PIIA default parameters

Interactions		Distance cut off in Å References	
H-Bond	DH…A (D=N, O, C; A=N,O)	dD-A ≤ 3.5	[4]
	SH…A (A=O, N)	dD-A ≤ 4.3	[13]
	DH…S (D=O, N, C)	dD-A ≤ 4.1	[13]
	SH…S	dD-A ≤ 4.5	[13]
	DH…π (D = O, N)	dD-A ≤ 4.3	[12]
	CH…π	dD-A ≤ 4.5	[11]
	SH…π	dD-A ≤ 4.5	[13]
Others	Disulfide Bridge	dS – S ≤ 2.4 Å	[2]
	Salt Bridge	d(+) – (–) ≤ 4.0 Å	[5]
	Hydrophobic interaction	dHPHOB–HPHOB ≤ 5.0 Å	[3]
	Non-bonded contacts	dATOM–ATOM ≤ 3.9 Å	[17]
	π–π	dπC – πC ≤ 7.0 Å	[6]
	Cation–π	d(+) – πC ≤ 6.0 Å	[7]
	Anion–π	d(–) – πC ≤ 6.0 Å	[8]
	Lone pair – π		
		N-π dN – πC ≤ 4.0 Å	[9]
		O-π dO – πC ≤ 4.0 Å	[9]
		S-π dS – πC ≤ 6.0 Å	[10]
